# Immediate efficacy of auricular acupuncture combined with active exercise in the treatment of acute lumbar sprains in 10 minutes: Protocol of a randomized controlled trial

**DOI:** 10.1371/journal.pone.0308801

**Published:** 2024-09-18

**Authors:** Xin Tang, Qifu Li, Gaoyangzi Huang, Xianmei Pei, Ziwen Chen, Ya Huang, Siwen Zhao, Taipin Guo, Zili Liu

**Affiliations:** 1 School of Second Clinical Medicine/The Second Affiliated Hospital, Yunnan University of Chinese Medicine, Kunming, Yunnan, China; 2 College of Acupuncture and Moxibustion and Tuina, Chengdu University of Traditional Chinese Medicine, Chengdu, Sichuan, China; The Affiliated Hospital of Zhejiang Chinese Medical University, CHINA

## Abstract

**Background:**

Acute lumbar sprain (ALS) is common musculoskeletal disorder characterized by severe low back pain and activity limitation, which significantly impacts the patient’s work and life. Immediate relief of pain and restoration of mobility in a short period of time are the main needs of patients when they visit the clinic. This study aims to evaluate the immediate efficacy of this combined treatment for ALS within 10 minutes.

**Methods:**

This is a single-center, prospective, randomized clinical trial. 128 eligible patients with ALS will be randomly allocated in a 1:1 ratio to either the auricular acupuncture (AA) group or the sham auricular acupuncture (SAA) group. All patients will receive a single 10-minute treatment. The primary outcome will be the change in pain intensity after 10 minutes of treatment. The secondary outcomes include changes in pain intensity at other time points (2, 5 minutes), changes in lumbar range of motion (ROM) at different time points, blinded assessment, treatment effect expectancy scale evaluation, and treatment satisfaction scale evaluation. All participants will be included in the analysis according to the intention-to-treat principle.

**Discussion:**

This is the first randomized controlled trial to assess the immediate efficacy of AA combined with active exercise for ALS. The findings of this study are expected to provide a simple and rapid treatment for ALS in clinical.

**Trial registration:**

Chinese Clinical Trial Registry ChiCTR2400083740. Registered 30 April 2024.

## Introduction

Acute lumbar sprain (ALS) is a common musculoskeletal disorder, often caused by the abrupt overstretching of the muscles, fascia, and ligaments in the lower back due to external forces [1]. It is characterized by severe pain and activity limitation in the lower back, which significantly impacts the patient’s work and life [2]. Therefore, quickly relieving symptoms in a short period of time is the main need of patients visiting the clinic.

According to the guidelines of the American College of Physicians, nonsteroidal anti-inflammatory drugs (NSAIDs) are recommended as the first-line pharmacological treatment for ALS, with diclofenac being the most commonly used NSAID [3,4]. A study has shown that diclofenac began to have effects at approximately 0.6 ± 0.05 hours after administration, with peak analgesia at 2.7 ± 0.24 hours [5]. Despite the convenience of oral administration, the delayed onset of action and moderate analgesic efficacy of NSAIDs limit their clinical application to some extent. Furthermore, there is guidance suggesting that non-pharmacological therapies can be used as a priority treatment, highlighting the importance of non-pharmacological intervention studies [6].

Auricular acupuncture (AA), as a form of traditional acupuncture therapy, offers potential advantages in pain management [7]. Due to its simple treatment modality and rapid onset of action, it is particularly suitable for acute pain disorders in clinical practice [8–10]. A previous study has shown significant pain relief with AA for lumbar pain, but limited efficacy for lumbar mobility disorders [11]. Exercise therapy, commonly utilized in the management of mobility disorders, has shown good efficacy for low back pain when combined with AA, significantly improving pain and function [12,13]. However, there is a lack of rigorous randomized controlled trials to support the combination of AA with active exercise in the treatment of ALS, and further studies are required.

This study aims to evaluate the immediate efficacy of AA combined with active exercise in the treatment of ALS in 10 minutes. The findings will provide a simple and rapid treatment for ALS in clinical.

## Materials and methods

### Trial strategy

**Fig 1:**
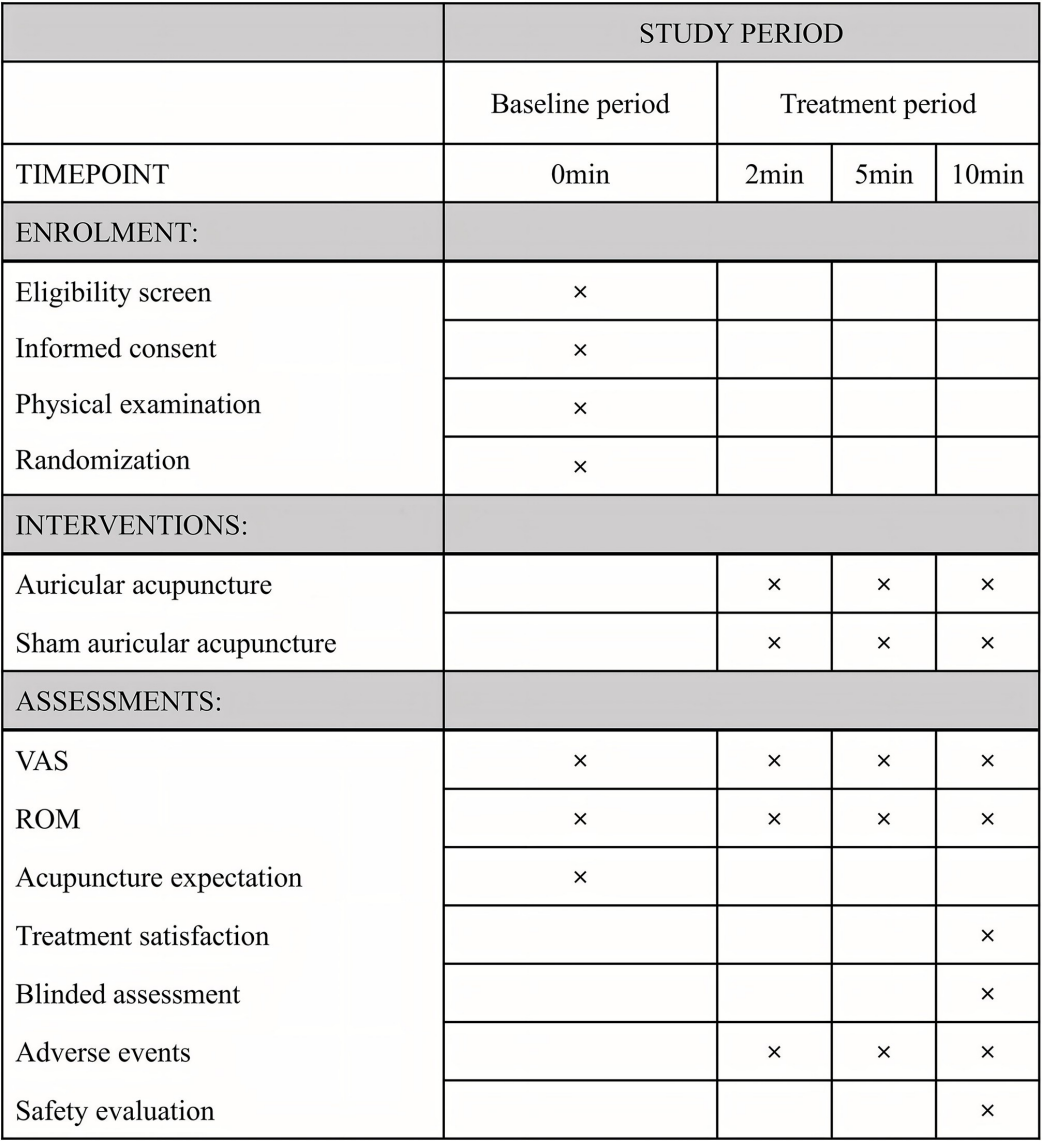
The SPIRIT schedule of enrollment, interventions, and assessments.

**Fig 2:**
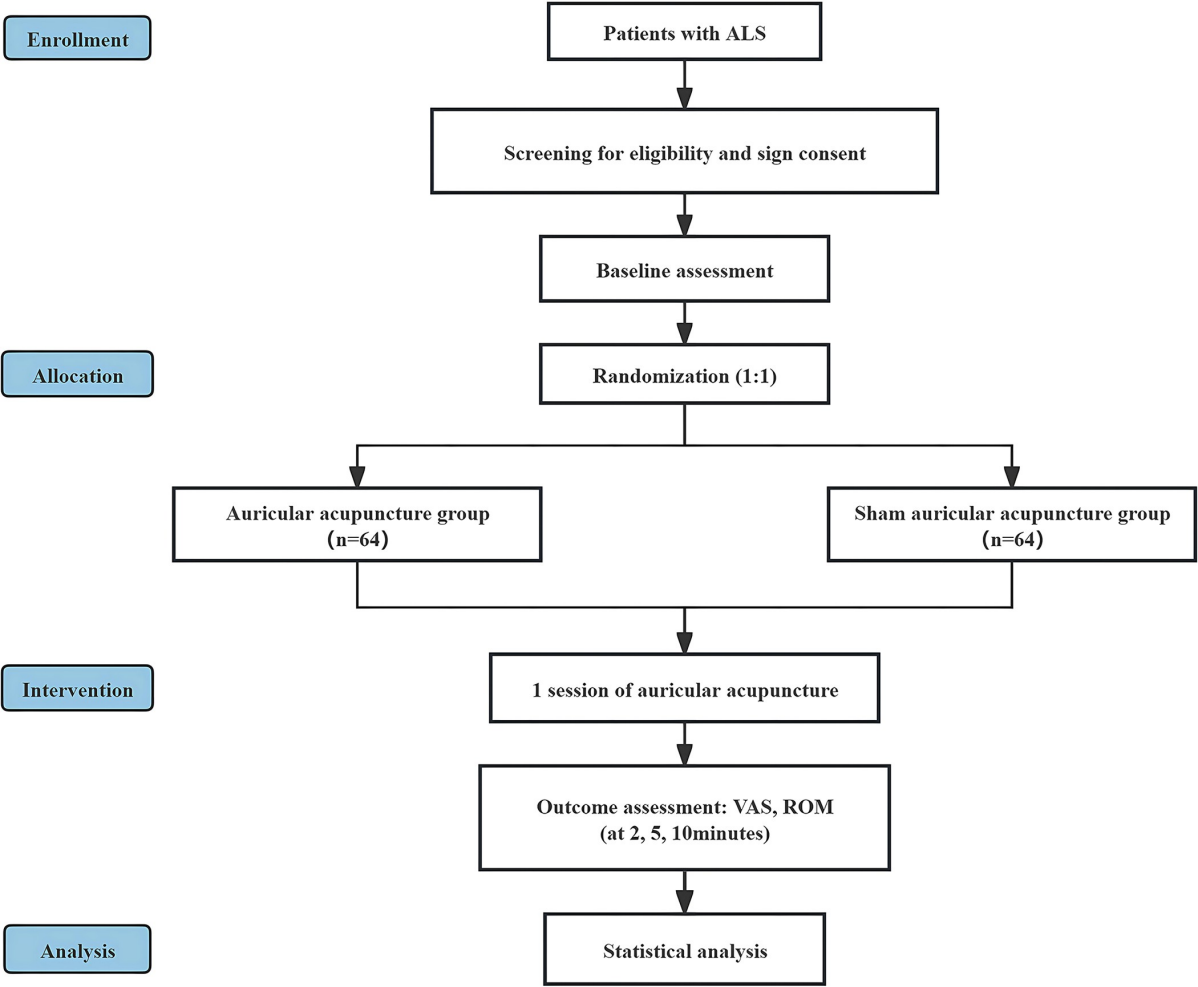
Flow chart of study design.

This is a randomized controlled trial to assess the immediate efficacy of AA combined with active exercise in ALS. The study will be conducted at the Second Affiliated Hospital of Yunnan University of Chinese Medicine, with a planned recruitment of 128 participants diagnosed with ALS (S1 Protocol). The schedule for enrollment, interventions and assessments is shown in Fig 1. The flow chart of the study design is shown in Fig 2. The study conforms to the Declaration of Helsinki and the Standard Protocol Items: Recommendations for Interventional Trials 2013 (SPIRIT 2013) (S1 File) [14]. The study protocol has been approved by the Ethics Committee (2024–024) (S2 File). The trial has been registered with the Chinese Clinical Trials Registry on April 30, 2024 (ChiCTR2400083740).

### Patients

Participants will be recruited from the acupuncture and orthopedics clinics of the Second Affiliated Hospital of Yunnan University of Chinese Medicine. An orthopedic surgeon with five years of clinical experience will strictly follow the inclusion and exclusion criteria to determine whether participants are eligible to participate in this study. Eligible participants will sign a written informed consent before the commencement of the study (S3 File). They can withdraw from the study at any time without penalty.

### Diagnostic criteria

ALS diagnosis is based on the "Clinical Diagnosis and Treatment Guidelines: Orthopedics" published by the Chinese Medical Association [15], which includes:

1. Definite trauma history and low back pain immediately after the injury;
2. Spasm of the lumbar muscles and limitation of movement;
3. Localized tenderness at the injury site;
4. No obvious abnormalities on imaging.

### Inclusion criteria

1. Meet the diagnostic criteria for ALS;
2. Unilateral low back pain, age 18–60 years;
3. Duration of the disease ≤ 3 days;
4. Moderate to severe pain, with the visual analogue score (VAS) between 4 and 8;
5. Signed the informed consent form.

### Excluded criteria

1. Combination of lumbar spondylolisthesis, lumbar spine tumor, fracture and other diagnosed definite pathological changes of the lumbar spine;
2. Low back pain caused by internal medicine diseases;
3. Coexistence of cardiovascular, hepatic, renal, pulmonary, and hematopoietic systems and other serious primary diseases;
4. Severe mental illness or intellectual disability, unable to cooperate with the completion of the questionnaire;
5. Women in pregnancy or breastfeeding;
6. Fear of acupuncture, or contraindications to acupuncture such as skin infection at the acupoint site;
7. Use of other analgesics within the past 6 hours.

### Dropped criteria

1. Participants who request study discontinuation or withdraw consent for study participation.
2. Participants who develop severe adverse reactions or complications, making it inappropriate to continue the treatment.

### Randomization, allocation and blinding

Participants will be randomly allocated into AA and sham auricular acupuncture (SAA) groups in a 1:1 allocation ratio. To mitigate selection bias, randomization sequences will be generated using SPSS version 28.0 (IBM, Chicago, IL) and concealed within opaque envelopes. Upon consenting to the random allocation principle, participants will select one of these envelopes to determine their group assignment. This allocation sequence number will then be meticulously documented in a Case Report Form (CRF) by a data administrator. To preserve the integrity of the study, blinding will be maintained for participants, researchers, outcome assessors, and statisticians regarding group assignments for the duration of the research. Due to the peculiarity of the acupuncture technique, the acupuncturist will not be blinded. However, the acupuncturist will not participate in subsequent outcome assessment and data analysis. To ensure unbiased results, they will be instructed not to disclose the participant’s allocation unless exceptional circumstances arise, such as severe infection or uncontrolled pain.

### Interventions

**Fig 3:**
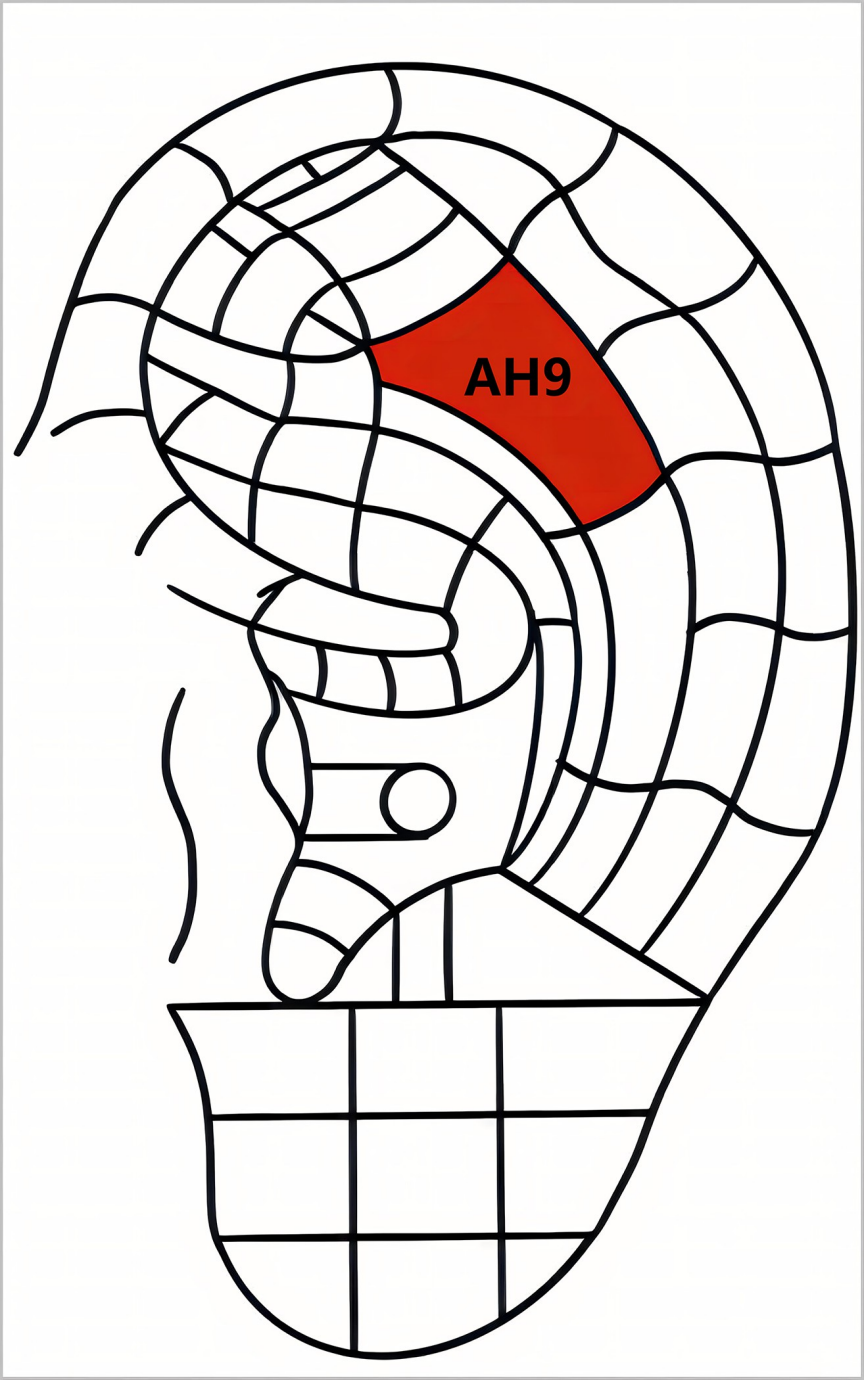
Location of acupoint.

The intervention measures will adhere to the Uniform Standard for Trial Reporting [16] and the Standard for Reporting Interventions in Clinical Trials of Acupuncture [17]. Auricular point selection is based on prior studies and traditional auriculotherapy, which involves selecting auricular regions that correspond to body anatomical parts. The Lumbosacral Vertebrae (AH9) (Located on the body of the antihelix posterior to the Abdomen) will be selected [18,19]. The auricular point will be positioned according to the World Federation of Acupuncture Societies Standard Acupoint Positioning [20], as depicted in Fig 3. The acupuncturist is a licensed practitioner with over three years of independent clinical experience.

### Auricular acupuncture group

**Fig 4:**
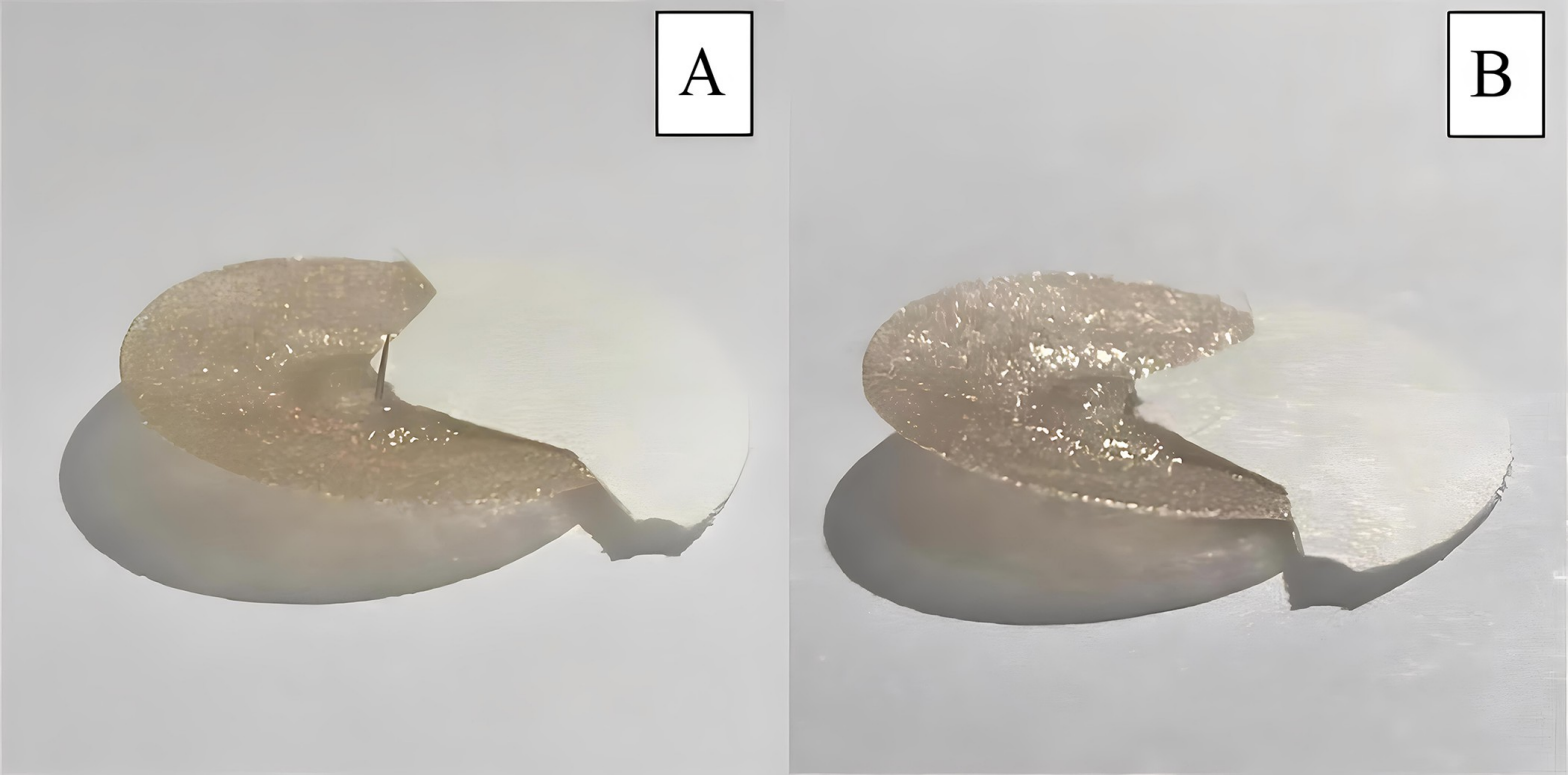
Auricular needle and Sham auricular needle.

Participants will be in a seated position. The acupuncturist will sterilize the skin of the healthy side AH9 with a 70% alcohol swab. Subsequently, a disposable auricular needle (0.2 mm in diameter, 1.5 mm in length, Seirin Corporation, Shizuoka, Japan) (Fig 4A) will be pierced vertically into the healthy side AH9, with a depth of 0.2 mm. After needle insertion, moderate pressure will be applied to elicit the "de qi" sensation. Upon achieving "de qi", participants will be asked to stand. The acupuncturist will stand behind the participant and support the participant’s waist. Then, the participant will be guided through moderate exercises, including forward bending, backward stretching, lateral bending, and rotation, all performed within the limits of their pain tolerance. The range and speed of these movements will gradually increase as the pain decreases. Each exercise will be performed 5 to 10 times, adjusted according to the participant’s tolerance [21].

### Sham auricular acupuncture group

This group will receive treatment with a placebo needle of the same shape (Fig 4B) which lacks a needle body and does not penetrate the skin but is merely taped at the acupoint. The treatment operation is the same as the AA group.

Participants in both groups will receive a single treatment session, with a 10-minute auricular needle retention time.

### Emergency treatment

If participants experience serious discomfort (such as pain, or fainting) during treatment, treatment will be stopped immediately. If a participant’s pain remains unalleviated or worsens after treatment, they will be given ibuprofen (Jiangsu Hengrui Medicine Co., Ltd., Lianyungang, Jiangsu, China), administered orally at a dose of 400 mg [22].

### Primary outcome

The primary outcome will be the change in pain intensity after the 10 minutes treatment as measured using VAS scores [23].

### Secondary outcomes

**Fig 5:**
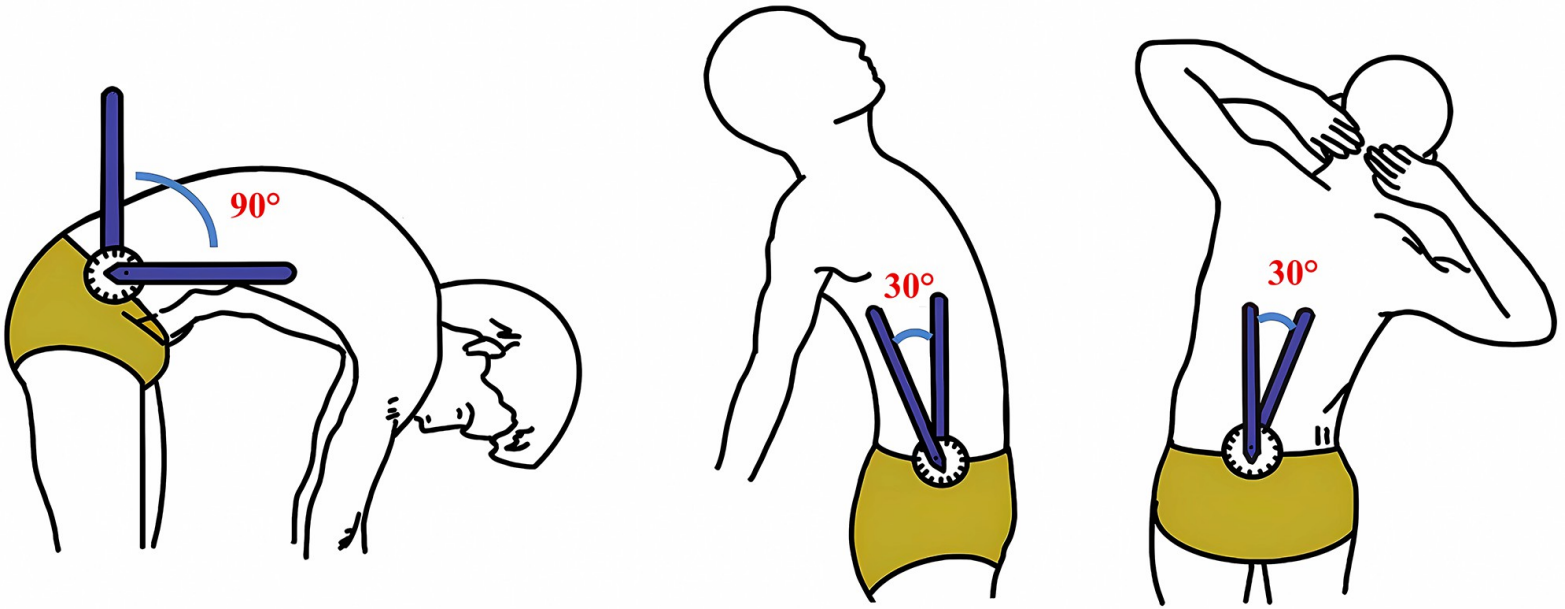
Lumbar motion measurement.

1. Changes in pain intensity at 2 and 5 minutes after treatment will be measured using VAS scores and will be compared between groups (S1 Table).
2. Lumbar range of motion (ROM) will be assessed at baseline, 2, 5, and 10 minutes during treatment. Participants will be instructed to stand with their feet slightly apart, shoulder width apart, and their body relaxed. Measurements will be taken for forward flexion, backward extension, right lateral flexion, and left lateral flexion in order [24,25], as shown in Fig 5 and S2 Table. These measurements will be compared between groups at different time points.
3. Patient expectations of treatment outcomes will be assessed at baseline using the efficacy expectations scale (S3 Table).
4. The patient’s satisfaction will be evaluated using a treatment satisfaction scale after the treatment (S4 Table).
5. The success of the blinding will be assessed at the end of treatment using a blinded questionnaire (S5 Table).

### Data management and confidentiality

The researchers will timely and carefully record preliminary participant data into Excel and anonymize using unique random identifiers to ensure confidentiality. The data collector will be responsible for data storage and management and will proofread the data rigorously. The Ethics Committee will review the trial regularly and supervise data processing. All raw clinical data will be retained for at least five years after publication. The Ethics Committee can adjust or terminate the trial. No conflict of interest between the committee and this study project.

### Adverse events and safety

This study will use AA and active exercise as intervention items. Any adverse events (AEs) observed during treatment will be fully evaluated and documented on a CRF. These AEs may include acupuncture-related events like severe localized pain, subcutaneous congestion, hematoma, localized infections, syncope, nausea, and unrelated symptoms such as cough or headache. All AEs will be treated promptly. Serious AEs will be reported to the study leader and and the Ethics Committee of the Second Affiliated Hospital of Yunnan University of Chinese Medicine within 48 hours.

### Sample size

This study is a superiority trial designed to assess whether AA combined with active exercise is more effective than SAA combined with active exercise for the treatment of ALS. A previous study showed that VAS scores decreased by 4.6 ± 1.0 and 2.7 ± 1.0 after 10 minutes of acupuncture combined with exercise and sham acupuncture combined with exercise for ALS, respectively [2]. Based on the results of this study, we predict that the change in VAS scores post-treatment will be 4.6 ± 1.0 in the AA group and 2.7 ± 1.0 in the SAA group, with α = 0.025 (unilateral, significance level), β = 0.1 (type II error rate, or power = 90%), Δ = 1.3 (minimum clinically important difference) [26], and K = 1 (allocation ratio). The sample size calculation is based on the following formula:

$$n_{c}\;=\;\frac{{\left(Z_{1-\alpha }+Z_{1-\beta }\right)}^{2}\sigma^{2}\left(1+\frac{1}{K}\right)}{{\left(\mu_{T}-\mu_{C}-\Delta \right)}^{2}}$$

It was calculated that a minimum of 58 participants were needed in each group [27]. Considering an 8 percent dropout rate, the adjusted sample size is 64 participants per group. Therefore, we plan to recruit at least 128 participants for this study.

### Statistical analysis

This study utilizes SPSS 28.0 for the analysis of clinical data. Continuous variables with normal distribution will be expressed as mean ± SD and continuous variables with non-normal distribution will be reported as median and interquartile range (IQR). For analyzing demographic data, continuous variables will be analyzed using the independent samples t-test or the Wilcoxon rank sum test depending on whether or not they conform to a normal distribution. Categorical variables will be analyzed using χ^2^ test or Fisher’s exact test. All significant demographic differences will be included as covariates in subsequent efficacy analyzes. The primary outcome indicator will be analyzed using analysis of covariance with baseline pain levels as a covariate and group as a factor. The secondary outcomes, including changes in pain intensity at other time points (2, 5 minutes) and ROM at different time points, will be analyzed using repeated measures ANOVA or Mann-Whitney U test, depending on the data’s distribution. Additionally, correlations between pain VAS scores and efficacy expectation scores will be explored using Spearman’s or Pearson’s correlation analysis. We will follow the intention-to-treat (ITT) principle and analyze all participants who receive treatment. Missing data, including from participants who withdraw or have their treatment stopped, will be processed using chained-equation multiple imputation [28]. The incidence of adverse events between different treatment groups will be compared using χ^2^ test or Fisher’s exact test.

### Trial status

The trial will commence recruitment and treatment on 1 May 2024 and is expected to be completed by 30 September 2026.

## Declarations

### Ethics approval and consent to participate

This study has been approved by the Ethics Committee of the Second Affiliated Hospital of Yunnan University of Chinese Medicine on 6 February 2024 (2024–024). The participants will sign a written informed consent before the start of the study. The results of this study will be published in peer-reviewed journals and presented at conferences.

## Discussion

ALS often presents with severe pain and limitation of movement in the lower back, necessitating immediate symptom improvement. The results of this study are expected to provide a rapid and effective non-pharmacological treatment for the clinical management of ALS.

The underlying pain mechanism of ALS involves sterile inflammatory responses triggered by mechanical pulling stimulation of the local muscle tissues, with inflammatory mediators stimulating nerves to induce pain [29]. Previous studies suggested that AA can rapidly reduce pain-related inflammation levels by activating the Peripheral Annexin A1-Formyl Peptide Receptor 2/ALX Pathway [30,31]. Moreover, AA can activate nociceptors, releasing neurotransmitters and endogenous substances (enkephalins and endorphins), thereby increasing pain thresholds and decreasing pain intensity [32]. ALS is usually accompanied by lumbar muscle spasms and embedded small joints in the lumbar spine, resulting in restricted movement [33]. Combined with exercise, the spastic and tense muscles can be relaxed, and the adhesive ligaments can be loosened, which can help to correct the embedded small joints and promote the recovery of dysfunction [34].

This study selects the auricular point corresponding to the lumbar region, AH9, which is the most commonly used for treating lower back disorders [35–37]. It has been shown that needling on the healthy side can awaken the gyrus area and inhibit the injurious signals on the affected side through a competitive mechanism, which is more effective than needling on the affected side in relieving pain and improving activity function [38–40]. ALS often usually occurs unilaterally, and we chose the AH9 of the healthy-side for treatment. Regarding the duration of treatment, previous studies of acute musculoskeletal conditions usually required 30 minutes or more of treatment [41], whereas recent evidence suggests that treatment of ALS can achieve significant effects in 10 minutes [1]. Given that ALS patients suffer from intolerable pain, they need relief in as short a time as possible. Therefore, this study set the observation time of 10 minutes, and measurements are taken again at 2 and 5 minutes to assess whether this combination therapy can achieve significant effectiveness in a shorter period of time. For outcome measurements, subjective pain assessed with the VAS and objective activity evaluated with the ROM will be chosen because they are clear and intuitive measures that facilitate rapid assessment [42,43]. Moreover, their direct correlation with the patient’s main symptoms ensures the reliability of the results [2].

This study is the first randomized controlled trial to assess the immediate efficacy of AA combined with active exercise therapy for ALS. The study outcomes will be assessed using a combination of subjective and objective indicators. The results of the study are expected to provide a simple, rapid, and effective treatment for the clinical management of ALS. However, some limitations need to be recognized. Firstly, due to the nature of the acupuncture intervention, acupuncturists cannot be blind to groupings. Secondly, this is a single-center study, limiting the generalisability of the trial findings to a wider population. Thirdly, this study did not set follow-up.

In summary, the main needs of patients with ALS during clinical visits are immediate pain relief and low back mobility improvement. AA combined with active exercise is considered a potential option for immediate relief. The findings of this study are expected to provide a simple, rapid, and effective treatment for the clinical management of ALS.

## Supporting information

S1 Table Visual analogue scale. (PDF)

S2 Table Range of motion. (PDF)

S3 Table Treatment expectations scale. (PDF)

S4 Table Treatment satisfaction scale. (PDF)

S5 Table Blinded questionnaire. (PDF)

S1 Protocol Study protocol with IRB approval. (PDF)

S1 File SPIRIT Fillable-checklist-15-Aug-2013. (PDF)

S2 File Ethical approval documentation. (PDF)

S3 File Informed consent of participants. (PDF)
