# Supplementary material for: Immediate efficacy of auricular acupuncture combined with active exercise in the treatment of acute lumbar sprains in 10 minutes: Protocol of a randomized controlled trial
Source: PLoS One. 2024 Sep 18;19(9):e0308801. doi: 10.1371/journal.pone.0308801 (PMC11410248; doi:10.1371/journal.pone.0308801)
Supplement: S1 Table — (PDF) [file pone.0308801.s001.pdf]

Visual analogue scale (VAS)

Use a 10cm VAS scale with a moving scale between 0 and 10 on the front and a number from 0 to 10 on the back, with 0 being no pain and 10 being the most painful. Please state your pain level according to the following scale.

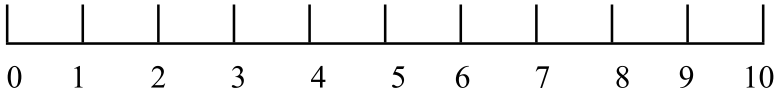

VAS scale (0-10 points)

0: no pain;1-3:light pain; 4-6:moderate pain; 7-10:severe and unbearable pain.

Projects	Before treatment	Treatment		
	0min	2min	5min	10min
Pain VAS score				
