# Supplementary material for: Immediate efficacy of auricular acupuncture combined with active exercise in the treatment of acute lumbar sprains in 10 minutes: Protocol of a randomized controlled trial
Source: PLoS One. 2024 Sep 18;19(9):e0308801. doi: 10.1371/journal.pone.0308801 (PMC11410248; doi:10.1371/journal.pone.0308801)
Supplement: S2 Table — (PDF) [file pone.0308801.s002.pdf]

Range of motion (ROM)

In this study, lumbar range of motion will be measured using an angulometer as follows:

The patient stands upright with feet shoulder-width apart and muscles relaxed. For flexion and extension of the lumbar spine, the center of the goniometer is fixed at the lateral line of the body, transverse to the 5th lumbar spine. For lumbar lateral flexion, the center of the goniometer is aligned with the 5th lumbar spinous process. The stationary arm is positioned vertically to the ground, and the moving arm parallels the line from the 7th cervical to the 5th lumbar vertebra's spinous processes. During flexion and extension measurements, the patient bends forward or extends backward slowly, keeping the knees straight. In lateral flexion measurements, with hands on the occipital protuberance, the patient bends to each side.

lumbar ROM		forward flexion	backward extension	left lateral flexion	right lateral flexion
Normal range (degree)		0°-90°	0°-30°	0°-30°	0°-30°
Before treatment	0min				
Treatment	2min				
	5min				
	10min				
