# Supplementary material for: Immediate efficacy of auricular acupuncture combined with active exercise in the treatment of acute lumbar sprains in 10 minutes: Protocol of a randomized controlled trial
Source: PLoS One. 2024 Sep 18;19(9):e0308801. doi: 10.1371/journal.pone.0308801 (PMC11410248; doi:10.1371/journal.pone.0308801)
Supplement: S4 Table — (PDF) [file pone.0308801.s004.pdf]

Treatment satisfaction scale

Did the treatment meet your initial expectations?	Yes☐ No☐
On a scale of 0 to 10, how satisfied are you with the treatment outcomes? (0 = not satisfied at all, 10 = completely satisfied).	__________
