# Supplementary material for: Immediate efficacy of auricular acupuncture combined with active exercise in the treatment of acute lumbar sprains in 10 minutes: Protocol of a randomized controlled trial
Source: PLoS One. 2024 Sep 18;19(9):e0308801. doi: 10.1371/journal.pone.0308801 (PMC11410248; doi:10.1371/journal.pone.0308801)
Supplement: S5 Table — (PDF) [file pone.0308801.s005.pdf]

Blinded questionnaire

Do you think you were given acupuncture treatment or placebo acupuncture?	Yes☐ No☐
How sure are you on your answer on a scale of 0 to 10? (0 = very uncertain and 10 = completely certain)	____________________
