# Supplementary material for: Immediate efficacy of auricular acupuncture combined with active exercise in the treatment of acute lumbar sprains in 10 minutes: Protocol of a randomized controlled trial
Source: PLoS One. 2024 Sep 18;19(9):e0308801. doi: 10.1371/journal.pone.0308801 (PMC11410248; doi:10.1371/journal.pone.0308801)
Supplement: S1 Protocol — (PDF) [file pone.0308801.s006.pdf]

Observational study on the immediate efficacy of auricular acupuncture combined with active exercise in the treatment of acute lumbar sprain

Study type:Clinical study

Study center:The Second Affiliated Hospital of Yunnan University of Chinese Medicine

Department:School of Second Clinical Medicine

Signature: Xin Tang

Version date: December 8, 2023

Contents

1. Background and purpose of the study 3

2. Study design and principles 4

3. Study population 6

4. Interventions 9

5. Observation indicators 11

6. Adverse event observation and analysis 13

7. Efficacy and safety evaluation 13

8. Explanation of scale evaluations 14

9. Quality control and assurance 16

10. Data management 18

11. Statistical analysis 20

12. Ethical principles 24

13. Summary and data preservation 28

1.Background and purpose of the study

Acute lumbar sprain (ALS) is a common musculoskeletal disorder, often caused by the abrupt overstretching of the muscles, fascia, and ligaments in the lower back due to external forces. It is characterized by severe pain and activity limitation in the lower back, which significantly impacts the patient's work and life. Therefore, quickly relieving symptoms in a short period of time is the main need of patients visiting the clinic.

According to the guidelines of the American College of Physicians, nonsteroidal anti-inflammatory drugs (NSAIDs) are recommended as the first-line pharmacological treatment for ALS, with diclofenac being the most commonly used NSAID. A study has shown that diclofenac began to have effects at approximately 0.6 ± 0.05 hours after administration, with peak analgesia at 2.7 ± 0.24 hours. Despite the convenience of oral administration, the delayed onset of action and moderate analgesic efficacy of NSAIDs limit their clinical application to some extent. Furthermore, there is guidance suggesting that non-pharmacological therapies can be used as a priority treatment, highlighting the importance of non-pharmacological intervention studies.

Auricular acupuncture (AA), as a form of traditional acupuncture therapy, offers potential advantages in pain management. Due to its simple treatment modality and rapid onset of action, it is particularly suitable for acute pain disorders in clinical practice. A previous study has shown significant pain relief with AA for lumbar pain, but limited efficacy for lumbar mobility disorders. Exercise therapy, commonly

utilized in the management of mobility disorders, has shown good efficacy for low back pain when combined with AA, significantly improving pain and function. However, there is a lack of rigorous randomized controlled trials to support the combination of AA with active exercise in the treatment of ALS, and further studies are required.

In this study, 128 patients with ALS will be recruited as study subjects, and sham auricular acupuncture (SAA) as control to observe the immediate efficacy of AA combined with active exercise for ALS.

2 Study design and principles

2.1 Study design

The study focuses on individuals with ALS, dividing them into the AA group and the SAA group. The aim is to study both intra-group (before and after treatment within each group) and inter-group comparisons (between the two groups).

2.2 Sample size

This study is a superiority trial designed to assess whether AA combined with active exercise is more effective than SAA combined with active exercise for the treatment of ALS. A previous study showed that VAS scores decreased by 4.6 ± 1.0 and 2.7 ± 1.0 after 10 minutes of acupuncture combined with exercise and sham acupuncture combined with exercise for ALS, respectively. Based on the results of this study, we predict that the change in VAS scores post-treatment will be 4.6 ± 1.0 in the AA group

and 2.7 ± 1.0 in the SAA group, with α=0.025 (unilateral), β=0.1, Δ=1.3, and K=1. The sample size calculation is based on the following formula:

$$n_c = \frac{\left(Z_{1-\alpha} + Z_{1-\beta}\right)^2 \sigma^2 \left(1 + \frac{1}{K}\right)}{(\mu_T - \mu_C - \Delta)^2}$$

It was calculated that a minimum of 58 participants were needed in each group. Taking into account the 8 percent dropout rate, we plan to recruit at least 128 participants for this study.

2.3 Randomization method

Participants will be randomly assigned to the AA group and the SAA group in a 1:1 ratio. To minimize selection bias, an independent statistician will use SPSS 28.0 (IBM, Chicago, IL, license code: f56b44b8d8e3562ad8a2) to generate random numbers. These numbers will be enclosed in opaque envelopes. Participants will choose one of these envelopes after agreeing to the principle of random allocation, which will determine their group assignment and the corresponding intervention method.

2.4 Blinding

Participants, outcome assessors, and statistical analysts will be blinded to group assignments. Although acupuncturists will be aware of the treatment allocations, they will not participate in the subsequent outcome assessment or data analysis.

2.5 Control group

The study establishes a SAA group as the control group.

2.6 Flowchart of the study procedure

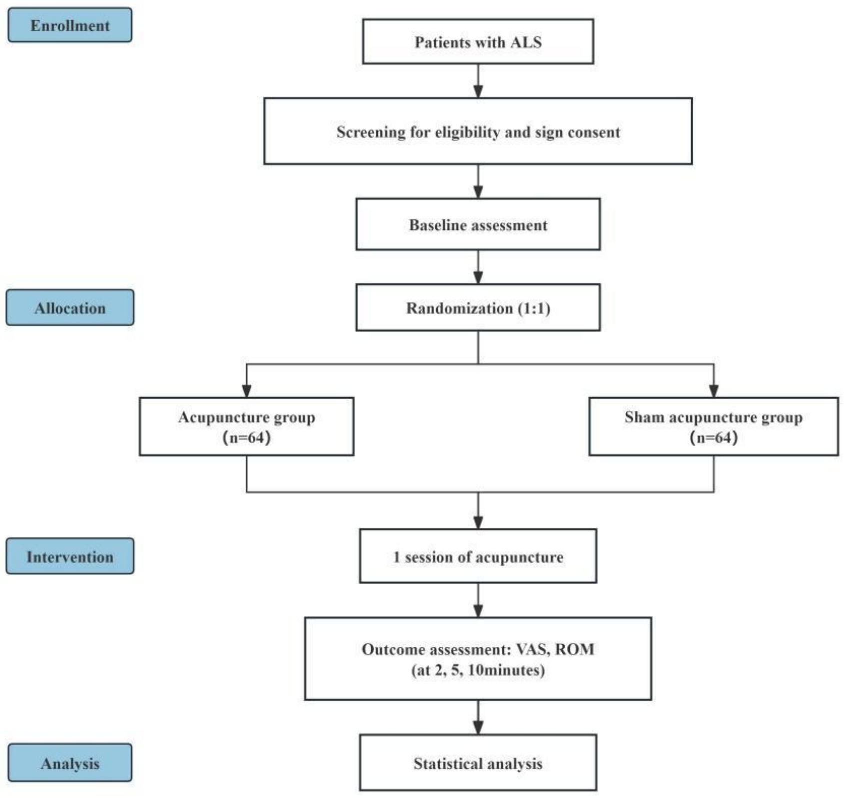

3 Study population

3.1 Participants with ALS

3.1.1 Diagnostic criteria

ALS diagnosis is based on the "Clinical Diagnosis and Treatment Guidelines: Orthopedics" published by the Chinese Medical Association, which includes:

1. Definite trauma history and low back pain immediately after the injury;
2. Spasm of the lumbar muscles and limitation of movement;
3. Localized tenderness at the injury site;
4. No obvious abnormalities on imaging.

3.1.2 Inclusion criteria

1. Meet the diagnostic criteria for ALS;

2. Unilateral low back pain, age 18-60 years;

3. Duration of the disease ≤ 3 days;

4. Moderate to severe pain, with the visual analogue score (VAS) between 4 and 8;

5. Signed the informed consent form.

3.1.3 Excluded criteria

(1) Combination of lumbar spondylolisthesis, lumbar spine tumor, fracture and other diagnosed definite pathological changes of the lumbar spine;

(2) Low back pain caused by internal medicine diseases;

(3) Coexistence of cardiovascular, hepatic, renal, pulmonary, and hematopoietic systems and other serious primary diseases;

(4) Severe mental illness or intellectual disability, unable to cooperate with the completion of the questionnaire;

(5) Women in pregnancy or breastfeeding;

(6) Fear of acupuncture, or contraindications to acupuncture such as skin infection at the acupoint site;

(7) Use of other analgesics within the past 6 hours.

3.1.4 Exclusion and dropout criteria

(1) Cases that do not meet the inclusion criteria and are erroneously included should be excluded.

(2) Subjects with poor compliance who withdraw themselves during the treatment.

(3) Cases that develop severe adverse reactions or complications, making it inappropriate to continue the treatment and thus terminating the trial.

3.1.5 Handling of excluded and dropped cases

(1) Upon a subject's dropout, the supervising physician should inquire about the patient's reasons, record the last data, and complete the assessments that can be completed.

(2) For cases withdrawing due to adverse reactions or ineffective treatment, the supervising physician should take appropriate measures based on the actual conditions of the subjects.

(3) Fill out the "Treatment Completion Summary" and "Clinical Trial Completion Status" in the Case Report Form (CRF).

(4) All excluded or dropped cases should be analyzed for intention-to-treat at the end of the trial.

(Note: Intention-to-treat analysis (ITT) refers to including subjects in the effectiveness analysis of the group they were assigned to, regardless of whether they received the group's intended treatment.)

3.1.6 Case termination criteria

(1) Subjects who experience increased pain or other severe adverse reactions during the study, making it inappropriate to continue participation.

(2) Subjects who request to withdraw from the clinical study midway.

(3) Patients who are uncooperative and non-compliant with treatment, despite repeated explanations by the clinician.

(4) Researchers should record in detail the reasons and timing of withdrawal from the study; cases that have surpassed half of the treatment duration should be included in

the efficacy analysis.

4 Interventions

4.1 Acupoint selection

Auricular point selection is based on prior studies and traditional auriculotherapy, which involves selecting auricular regions that correspond to body anatomical parts. The Lumbosacral Vertebrae (AH9) (Located on the body of the antihelix posterior to the Abdomen) will be selected. The auricular point will be positioned according to the World Federation of Acupuncture Societies Standard Acupoint Positioning, as depicted in Fig 1.

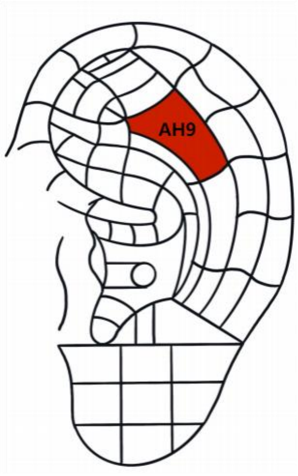

Fig 1 Location of acupoint

4.2 Appliance selection

(1) Auricular needles: Use Seirin Corporation's single-use sterile microneedles, registration number: National Food and Drug Supervision and Medical Equipment (Import) No. 2012-2272550. Specifications are 0.2 × 1.5 mm, see Figure 2A.

(2) Sham auricular needles: Same Seirin Corporation single-use sterile microneedles, registration number as above. Placebo needles look identical to real needles but lack a needle body and do not penetrate the skin, see Figure 2B.

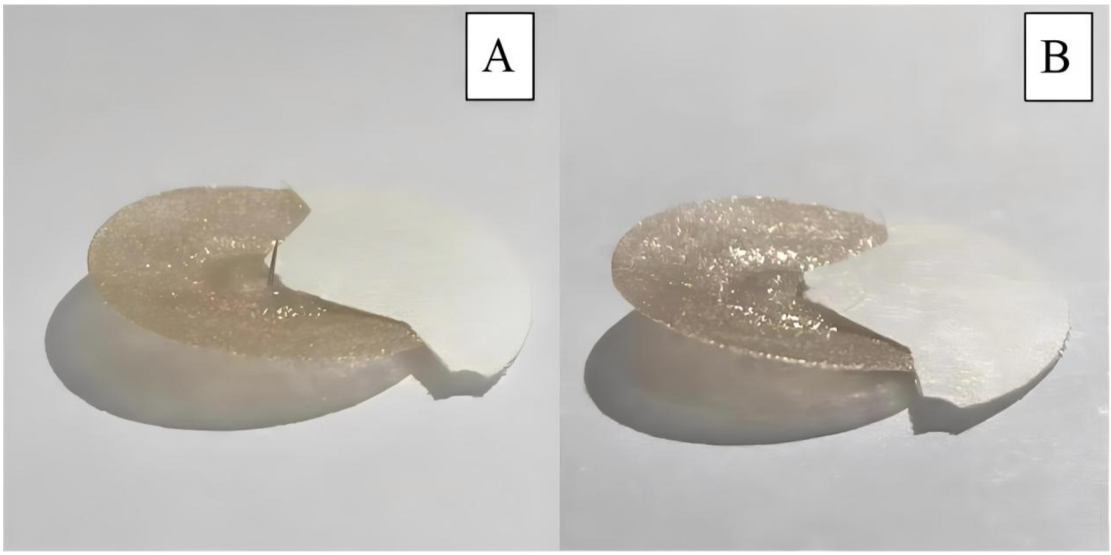

Fig 2. Auricular needle and Sham auricular needle

4.3 Procedures

(1) Auricular acupuncture group: Participants will be in a seated position. The acupuncturist will sterilize the skin of the healthy side AH9 with a 70% alcohol swab. Subsequently, a disposable auricular needle will be pierced vertically into the healthy side AH9. After needle insertion, moderate pressure will be applied to elicit the "de qi" sensation. Upon achieving "de qi", participants will be asked to stand. The acupuncturist will stand behind the participant and support the participant's waist. Then, the participant will be guided through moderate exercises, including forward bending, backward stretching, lateral bending, and rotation, all performed within the limits of their pain tolerance. The range and speed of these movements will gradually increase as the pain decreases.

(2) Sham auricular acupuncture group: This group will receive treatment with a placebo needle of the same shape which lacks a needle body and does not penetrate the skin but is merely taped at the acupoint. The treatment operation is the same as the AA group.

Participants in both groups will receive a single treatment session, with a 10-minute auricular needle retention time.

4.4 Remedial pain management

If patients experience intolerable lower back pain that does not improve post-treatment, they will take the non-prescription NSAID pain reliever ibuprofen. The dosage, time of administration, and the duration until pain relief are recorded.

4.5 Observation period

Baseline assessments will be performed before enrollment, and pain intensity and ROM evaluations will be conducted 2, 5, and 10 minutes post-treatment.

5 Observation indicators

5.1 General items

Patient's name, gender, age, diagnosis, and course of illness will be recorded in the patient CRF.

5.2 Biological indicators

(1) Demographic signs: Gender, age, height, weight, occupation, marital status, and educational background.

(2) Vital signs: Body temperature, heart rate, breathing.

(3) Basic medical history and routine physical examination.

These indicators will be recorded at the time of enrollment.

5.3 Diagnostic indicators

Chief complaints and clinical manifestations, medical history, physical examination; auxiliary examinations such as lumbar spine X-rays, CT, MRI will be conducted for disease diagnosis by the primary physician.

5.4 Measurement indicators

5.4.1 Primary outcome

The primary outcome will be the change in pain intensity from baseline to 10 minutes after treatment as measured using VAS scores.

5.4.2 Secondary outcomes

(1) Changes in pain intensity at 2 and 5 minutes after treatment will also be measured using VAS scores.

(2) Lumbar range of motion (ROM) will be assessed at baseline, 2, 5, and 10 minutes during treatment. Participants will be instructed to stand with their feet slightly apart, shoulder width apart, and their body relaxed. Measurements will be taken for forward flexion, backward extension, right lateral flexion, and left lateral flexion in order.

(3) Patient expectations of treatment outcomes will be assessed at baseline using the efficacy expectations scale.

(4) The success of the blinding will be assessed at the end of treatment using a blinded questionnaire.

6 Adverse event observation and analysis

All adverse events occurring during the study must be recorded and reported, and participants must be treated promptly and appropriately. Researchers will inform participants and ask them or their families to accurately report any changes in their condition post-treatment. Physicians should avoid leading questions. During efficacy observations, attention must be paid to adverse events. If incidents such as fainting, pain at the needle site, bleeding, hematoma, or infection occur during acupuncture, these will be recorded and appropriately handled until full recovery. Regardless of whether the adverse event is related to the treatment method, detailed records must be kept including the time of occurrence, symptoms, signs, severity, duration, lab test indicators, methods and results of treatment, course, follow-up times, and an analysis of the causes of adverse events.

7 Efficacy and safety evaluation

7.1 Overall efficacy evaluation of participants

(1) After completing all treatments, the effectiveness is assessed based on the "Traditional Chinese Medicine Syndrome Diagnosis and Treatment Standards" (2022 Edition) and improvements in patient observation indicators.

(2) After treatment, participants evaluate the effects of the acupuncture treatment.

7.2 Safety evaluation

7.2.1 Adverse events

Record any adverse events (names, symptoms) that occur during different treat

ment regimens, including the time of occurrence, severity, measures taken, and outcomes, to assess their relevance to the treatment.

7.2.2 Safety assessment

Statistically compare adverse events that occur under different treatment regimens in this study and evaluate the safety of each regimen. Thoroughly document the time of occurrence, symptoms, severity, measures taken, and resolution of adverse events. In cases of adverse events, clinical physicians will decide whether to discontinue the trial based on the patient's condition, with severe cases being reported to the Medical Ethics Committee of the Second Affiliated Hospital of Yunnan University of Chinese Medicine within 24 hours, while also making the appropriate records.

8 Explanation of scale evaluations

8.1 Visual analogue scale

Use a 10cm VAS scale with a moving scale between 0 and 10 on the front and a number from 0 to 10 on the back, with 0 being no pain and 10 being the most painful. Please state your pain level according to the following scale.

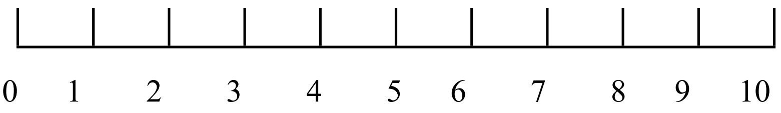

VAS scale (0-10 points)

0: no pain;1-3:light pain; 4-6:moderate pain; 7-10:severe and unbearable pain.

Projects	Before treatment	Treatment		
	0min	2min	5min	10min

Pain VAS score				

8.2 Range of motion

In this study, lumbar range of motion will be measured using an angulometer as follows:

The patient stands upright with feet shoulder-width apart and muscles relaxed. For flexion and extension of the lumbar spine, the center of the goniometer is fixed at the lateral line of the body, transverse to the 5th lumbar spine. For lumbar lateral flexion, the center of the goniometer is aligned with the 5th lumbar spinous process. The stationary arm is positioned vertically to the ground, and the moving arm parallels the line from the 7th cervical to the 5th lumbar vertebra's spinous processes. During flexion and extension measurements, the patient bends forward or extends backward slowly, keeping the knees straight. In lateral flexion measurements, with hands on the occipital protuberance, the patient bends to each side,as depicted in Fig 3.

lumbar ROM		forward flexion	backward extension	left lateral flexion	right lateral flexion
Normal range (degree)		0°-90°	0°-30°	0°-30°	0°-30°
Before treatment	0min				
Treatment	2min				
	5min				
	10min				

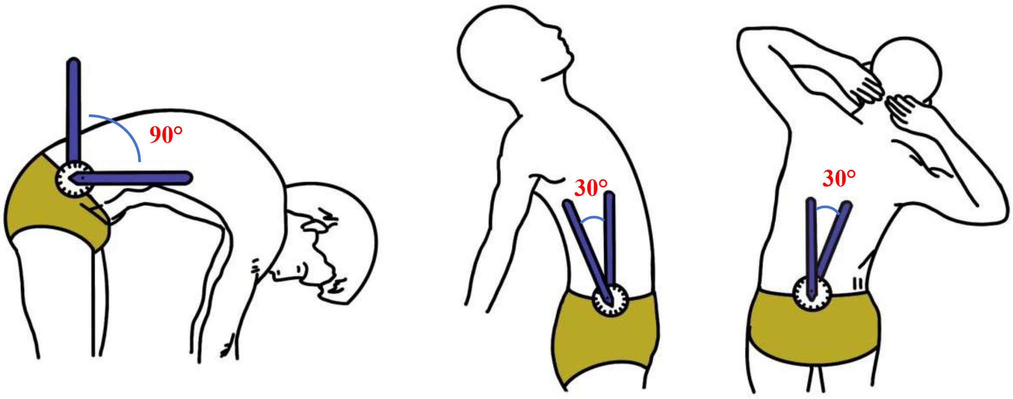

Fig 3 Lumbar motion measurement

9 Quality control and assurance

9.1 Analysis of study influencing factors

9.1.1 Analysis

(1) Selection Bias: To avoid selection bias, the most effective measure is the use of a randomized control design, especially the random assignment of subjects into groups to ensure balanced and comparable groups. This prevents researchers from arbitrarily deciding group assignments or treatments based on subjective desires, thus avoiding biased outcomes.

(2) Measurement Bias: When measuring outcomes between AA and SAA groups, especially when it's known which group is which, measurement observations are likely to differ due to subjective reasons, leading to judgment biases, particularly in non-blinded observations.

(3) Confounding Bias: Confounding bias mainly appears during the result analysis phase, where two factors may be intertwined or affect each other, possibly attributing

the cause of a result incorrectly due to other co-existing factors (confounders) that could produce the same outcome, leading to exaggerated or minimized conclusions.

9.1.2 Control

(1) Choice of Study Design: This study uses a randomized control design, which is the most effective way to avoid selection bias.

(2) Strict Selection of Subjects: Strict inclusion criteria are one of the important methods to control bias. Establishing clear inclusion and exclusion criteria restricts study subjects to a specific range, reducing differences and facilitating objective conclusions about observed factors.

(3) Blinded Measurement and Judgment: Blinding is the most effective way to avoid measurement biases by researchers and subjects. If the researcher knows which treatment a participant is receiving, they might unconsciously favor reporting improvements in the treatment group. Although researchers may be honest, they tend to hope for favorable outcomes, thus the best way to avoid this bias is through blinded measurements and judgments. Although acupuncturists will be aware of the treatment allocations, they will not participate in the subsequent outcome assessment or data analysis. Blinded statistical analysis during the data summarization stage to ensure the reliability of the study results.

9.2 Research training

To ensure the smooth conduct of the study, a specialized clinical training session will be held before the start of the study. All researchers will receive uniform training on the implementation plan and detailed operational training to familiarize and master

the research process and specific implementation rules, enhancing internal consistency among researchers and between observers, and ensuring the reliability of research conclusions.

9.3 Measures to improve participant compliance

To achieve better compliance, ensuring that the number of cases analyzed at the end is no less than 80% of those entering the study, the following measures will be used: adhere to the principle of voluntariness, sign informed consent forms with participants and their families; improve and maintain good compliance through medical quality, clinic environment, and medical costs, encouraging participants to adhere to the treatment during the study; establish good doctor-patient relationships, explain the condition, treatment purposes and necessity in detail, and obtain consent and cooperation from participants. Communicate with patients before starting treatment, observe the efficacy after one treatment session, and if the subsequent relief is not significant, offer two additional acupuncture treatments free of charge. At the end of the treatment, review and perfect the relevant records together with the participants.

9.4 Quality control and assurance system

The project leader will supervise and inspect the entire study process, ensuring all study data records, reports, and case report form entries are true, accurate, and complete, and consistent with the original data.

10 Data management

Researchers are responsible for data management of the project, with supervision by

the project leader.

10.1 Definition of original data

(1) All original records entered in the case report forms;

(2) All original data from laboratory tests and other examination reports.

10.2 Data recording

Researchers must fill in clinical research records promptly, accurately, completely, in a standardized manner, and truthfully.

(1) All cases must be documented and CRFs filled in completely as per the study protocol, with no fields left blank (use a diagonal line for any spaces without records);

(2) Medical records and CRFs, as original records, can only be corrected by striking through, annotating the revised data with reasons, and must be signed and dated by the clinician and researcher involved in the clinical study; no erasing or covering of original records is allowed;

(3) Data significantly above or beyond clinically acceptable ranges must be verified, with necessary explanations provided by the attending clinician;

(4) After the treatment observation of each participant is complete, researchers must submit the case report forms and medical records to the principal investigator for review and signature within one week.

10.3 Data verification

(1) The principal investigator must periodically verify that researchers are following the study protocol, checking participants' informed consent and screening inclusion;

(2) Confirm all case report forms are filled in promptly and correctly and consistent

with original data; all errors or omissions are corrected or noted, signed and dated by the researchers; any changes in treatment alterations, concomitant medications, complications, or missed examinations must be confirmed and recorded;

(3) Ensure that reasons for participant withdrawals are noted in the case report forms; all adverse events, especially serious adverse events, must be reported and recorded within the specified time.

11 Statistical analysis

Clinical data part will be handled by Yunnan University of Traditional Chinese Medicine, using SPSS for statistical analysis.

11.1 Statistical analysis content and methods

11.1.1 Statistical analysis sets

11.1.1.1 Efficacy evaluation data set

(1) Full Analysis Set (FAS): Includes all randomized participants. For cases not observed throughout the entire treatment process, the last observed outcome (last observation carry-forward: LOCF) is carried forward to the point of data missing. The number of participants evaluated for efficacy at the endpoint is consistent with the number at the start of the study. Comprehensive efficacy analysis is conducted using the Per Protocol Set (PPS) and the Full Analysis Set.

(2) Per Protocol Set (PPS): Includes participants who complied with the study treatment protocol, primarily those who can be measured for key indicators and have no major violations of the study protocol. Analyses of demographic and other baseline characteristics and other efficacy indicators are conducted using the PPS.

11.1.1.2 Safety data set

Safety Data Set (SS): All participants who completed treatment after randomization.

11.1.2 Participant handling

Summarize the number of participants who started, completed, and discontinued the study. Additionally, summarize the reasons for discontinuation.

11.1.3 Demographics and baseline characteristics

(1) Demographic Indicators: Age, height, and weight, etc.

(2) Basic Vital Signs: Resting heart rate, breathing, blood pressure, and body temperature, etc.

11.1.4 Efficacy evaluation

All efficacy evaluation data will be summarized separately according to the FAS and PPS sets, with the FAS set as the primary analysis method.

11.1.5 Statistical analysis methods

This study utilizes SPSS 28.0 for the analysis of clinical data. Continuous variables with normal distribution will be expressed as mean ± SD and continuous variables with non-normal distribution will be reported as median and interquartile range (IQR). For analyzing demographic data, continuous variables will be analyzed using the independent samples t-test or the Wilcoxon rank sum test depending on whether or not they conform to a normal distribution. Categorical variables will be analyzed using χ^2 test or Fisher's exact test. All significant demographic differences will be included as covariates in subsequent efficacy analyzes. The primary outcome indicator will be analyzed using analysis of covariance with baseline pain levels as a

covariate and group as a factor. The secondary outcomes, including changes in pain intensity at other time points (2, 5 minutes) and ROM at different time points, will be analyzed using repeated measures ANOVA or Mann-Whitney U test, depending on the data's distribution. Additionally, correlations between pain VAS scores and efficacy expectation scores will be explored using Spearman's or Pearson's correlation analysis. We will follow the intention-to-treat (ITT) principle and analyze all participants who receive treatment. Missing data of withdrawn participants will be processed using chained-equation multiple imputation.

(1) Case Distribution: Size of different data sets in each group, distribution of cases across centers, detailed list of dropouts, discontinuations, and reasons.

(2) Comparability Analysis: Compare demographic data and other baseline values to assess comparability between groups.

(3) Compliance Analysis: Compare whether patients in each group followed the design protocol. Compliance is evaluated based on the records in the participant's CRF form, calculating percentages for <80%, 80%–100%, and >100% compliance.

(4) Factors Affecting Efficacy Analysis: If there are significant differences between groups before the study or factors significantly affecting efficacy, these factors should be considered as covariates in the group comparisons, requiring covariance analysis or logistic regression, and a detailed list of concomitant medication use.

(5) Safety Analysis: Firstly, list and describe adverse events in each group according to the relatedness requirements (including the number and incidence of various adverse events), and list their causes and explanations. If comparison is necessary,

chi-square tests may be used for statistical analysis of adverse events, summarized according to safety evaluation criteria, which are graded as follows:

1. Level 1: Safe, no adverse reactions;

2. Level 2: Relatively safe, with mild adverse reactions that do not require intervention and can continue treatment;

3. Level 3: Safety concerns, with moderate adverse reactions that can continue treatment after interventions;

4. Level 4: Study discontinued due to adverse reactions.

11.1.6 Adverse events

All adverse events (AEs) occurring during the study must be recorded and reported, and timely and appropriate measures must be taken for participants. Researchers must inform participants to honestly report changes in their condition after treatment, avoiding leading questions. During the observation of efficacy, pay attention to adverse events. If adverse events such as fainting, pain at the acupuncture site, bleeding, hematoma, or infection occur during acupuncture, participants will receive recorded and timely proper treatment until fully recovered.

Regardless of whether adverse events are related to the treatment method of the study, they must be thoroughly recorded, including the time of occurrence, symptoms, signs, severity, duration, laboratory indicators, treatment methods and outcomes, course, follow-up time, etc., and analyze the causes of adverse events.

(1) Serious Adverse Event Determination: Serious Adverse Events (SAEs) refer to unforeseen clinical events occurring during the study period, including patient death,

life-threatening situations, resulting in hospitalization, prolonging hospital stay, long-term or significant functional impairment/disability, or requiring medical or surgical treatment to prevent serious consequences.

(2) Handling of Adverse Events: AEs occurring during the study will be summarized by total number, system organ class (SOC), or other terms, summarizing at least once occurring event incidence. AEs will also be summarized by severity and relatedness to acupuncture. SAEs leading to study discontinuation will also be summarized.

For each count and incidence, each participant is counted only once regardless of the number of occurrences. When summarizing adverse events, missing severity ratings or causality assessments will be classified as "serious" and "definitely related."

All AEs will be presented in lists, including SAEs leading to study discontinuation due to acupuncture, and events leading to fatal outcomes. Missing values and incomplete dates in the lists will be displayed consistent with original records.

12 Ethical principles

12.1 Ethical review

This study project must be reviewed and approved by the institutional ethics committee before the clinical study can commence.

12.2 Benefits and risks

Participants in this study project will be informed about the content of the clinical study, including its purpose, methods, and procedures such as treatment methods, grouping, and testing items. During the study, participants will receive AA or SAA

and undergo free examinations related to the project. Adverse events, such as fainting, pain at the acupuncture site, bleeding, hematoma, or infection during acupuncture, will be recorded and properly addressed until fully resolved.

Participants' personal data must be kept confidential. Participation in the clinical study is completely voluntary, and participants can withdraw at any stage without facing discrimination or retaliation, and their medical treatment and rights will not be affected. If any damage related to the clinical study occurs, participants will be entitled to appropriate compensation.

12.3 Informed consent

(1) Researchers must familiarize themselves with and verify the comprehensiveness and compliance of the informed consent form (including ethical committee approval and adherence to the rights and duties of participants as stipulated in the Declaration of Helsinki) before discussing with participants and their families. Any amendments to written materials or the informed consent form must be approved by the ethics committee before re-obtaining informed consent from participants.

(2) The informed consent document should be explained to participants in clear and understandable language, including the purpose of the study, procedures, duration, examinations, expected benefits, and potential risks. Efforts should be made to avoid technical jargon to ensure participants fully understand the consent content.

(3) Participation in the study is voluntary, and participants have the right to withdraw at any time without discrimination or retaliation, and their medical treatment and rights will not be affected.

(4) All personal data of participants involved in the study are confidential.

(5) Participants may be assigned to different groups within the study.

(6) Participants must be informed that the clinical trial treatment is provided free of charge and has been tested and approved. In case of study-related harm, participants are entitled to treatment and compensation. Participants can lodge complaints with the hospital's clinical research office or the ethics committee if they have concerns about the study process.

(7) Participants and their families should be given sufficient time and opportunity to ask about the study details and any other related questions, ensuring they have fully understood the information before volunteering without coercion, undue pressure, or inducement.

(8) The informed consent form should be signed and dated by the participant and their family members; for participants who are unable to perform legally relevant actions, their legal representative (including family members) must sign and date the form.

(9) The researcher or their representative must also sign and date the informed consent form, indicating that they have explained the steps of the study and the potential risks and benefits to the participant.

(10) If necessary (e.g., if the participant and their legal representative are illiterate), a witness should be present throughout the informed consent process. After a detailed explanation of the informed consent document, the witness should sign and date the form to confirm that the verbal consent matches the written consent.

(11) The signing of the informed consent should occur before any research procedure

is performed. If the consent cannot be signed before the study due to justifiable reasons, it should be signed as soon as possible, and the form should note the date and explanation for any amendments.

(12) Two copies of the signed and dated informed consent form should be made; one copy is given to the participant or their legal representative, and the other is kept by the researchers for archiving.

12.4 Source of participants

All participants with ALS in this study will be recruited from the outpatient department of the Second Affiliated Hospital of Yunnan University of Chinese Medicine.

12.5 Medical care and protection of participants

In accordance with the Declaration of Helsinki, the informed consent document is drafted in a language and script that the participant or their legal representative/guardian can understand. It details the purpose, methods, and processes of the study, including treatment measures, grouping, testing, potential benefits, and risks, as well as alternative diagnostic and treatment options available. It also ensures that participants' personal data are confidential and that their participation is completely voluntary. Participants have the right to withdraw from the study at any stage without discrimination or retaliation, and their medical treatment and rights will not be affected. If any damage related to the clinical study occurs, participants will be entitled to appropriate compensation. Only after obtaining the signatures and dates from the participant and their legal representative/guardian can the clinical study

proceed. If the participant and their representative are illiterate, a witness must be present, and after a detailed explanation and oral consent, the witness must sign and date the consent form. Researchers must rigorously implement the informed consent process to ensure that participants fully understand the study requirements and cooperate with the research.

13 Summary and data preservation

Upon completion of the research project, a summary of the data will be made, and original materials such as the research protocol, informed consent forms, ethical committee approvals, ethical committee member list, completed and signed case report forms with dates, original serious adverse event reports, CRF forms, operation manuals, researcher's signature stamps, and all related original materials will be archived by the research team.
