# Supplementary material for: Immediate efficacy of auricular acupuncture combined with active exercise in the treatment of acute lumbar sprains in 10 minutes: Protocol of a randomized controlled trial
Source: PLoS One. 2024 Sep 18;19(9):e0308801. doi: 10.1371/journal.pone.0308801 (PMC11410248; doi:10.1371/journal.pone.0308801)
Supplement: S2 File — (PDF) [file pone.0308801.s008.pdf]

云南中医药大学第二附属医院医学伦理委员会

Second Affiliated Hospital of Yunnan University of Chinese Medicine Ethics Committee

临床试验批准函

Approval Letter

云中二附院伦审【2024-024】号

项目名称	耳针结合主动运动治疗急性腰扭伤的即时疗效观察研究		
项目类型	☐药物临床试验 ☐医疗器械临床试验 ☐临床科研项目 ☑研究者自发项目 ☐医疗新技术 ☐其他		
药物/器械名称		剂型/器械类型	
研究方法	☐实验性研究 ☐观察性研究 （ ☐回顾性 ☐前瞻性 ） ☑其他		
申办单位	云南中医药大学第二附属医院		
组长单位	云南中医药大学第二附属医院		
参加单位			
承担科室	针灸科	主要研究者	郭太品
审查方式	☑会议审查 ☐快速审查	审查日期	2024 年 2 月 6 日

审查后批准的文件

1. 临床研究方案
2. 知情同意书
3. 招募招募受试材料
4. 病例报告表
5. 研究者手册
6. 应急预案
7. 研究者履历及研究团队成员

审查意见

同意

跟踪审查频率	☐3 个月 ☐6 个月 ☑12 个月 ☐其他______		
有效期	2025 年 2 月 6 日		
联系人	陈柏君	联系电话	13888244951
主任委员签名	袁[illegible]		
伦理委员会	（盖章）		
日期	2024 年 2 月 6 日		

云南中医药大学第二附属医院伦理委员会

Second Affiliated Hospital of Yunnan University of Chinese Medicine Ethics Committee

Approval Letter

Yunnan University of Chinese Medicine Second Affiliated Hospital Ethics Review【2024-024】

Project Title	Observational study on the immediate efficacy of auricular acupuncture combined with active exercise in the treatment of acute lumbar sprain		
Project Type	☐Drug clinical trial ☐Medical device clinical trial ☐Clinical research project ☑Investigator-initiated project ☐New medical technology ☐Others		
Drug/Device Name		Dosage Form/Device Type	
Research Method	☐Experimental study ☐Observational study (☐Retrospective ☐Prospective) ☑Others		
Sponsor	Second Affiliated Hospital of Yunnan University of Chinese Medicine		
Leading Unit	Second Affiliated Hospital of Yunnan University of Chinese Medicine		
Participating Units			
Responsible Department	Acupuncture department	Principal Investigator	Guo Taipin
Review Method	☑Committee review<br>☐Expedited review	Review Date	February 6, 2024
Approved documents post-review			

1. Clinical research protocol
2. Informed consent form
3. Recruitment materials
4. Case report form
5. Investigator's brochure
6. Emergency plan
7. Investigator and research team members' CVs

Review comments			
Approved			
Follow-up Review Frequency	☐3 months ☐6 months ☑12 months ☐Others		
Validity Period			
Contact Person	Chen Baijun	Contact Number	13888244951
Signature of the Chairman	Yuan Kai		
Ethics Committee			
Date	February 6, 2024		
