# Supplementary material for: Immediate efficacy of auricular acupuncture combined with active exercise in the treatment of acute lumbar sprains in 10 minutes: Protocol of a randomized controlled trial
Source: PLoS One. 2024 Sep 18;19(9):e0308801. doi: 10.1371/journal.pone.0308801 (PMC11410248; doi:10.1371/journal.pone.0308801)
Supplement: S3 File — (PDF) [file pone.0308801.s009.pdf]

Informed Consent

Informed Page

Name of Project: Clinical study of the immediate efficacy of auricular acupuncture combined with active exercise in the treatment of acute lumbar sprains.

Source of project: This study is supported by the "Liang Fanrong Expert Workstation" of Yunnan Province-Yunnan Science and Technology Programme (202305AF150072), the Yunnan Ten Thousand Talents Plan Youth Project (YNWR-QNBJ-2019-257) and the "Liu Zili Famous Doctor" special talent program of the Yunnan Provincial Xing Dian Talent Support Program (Yunnan Party Talent Office 2022 No.18).

Project research organization: School of Second Clinical medicine/The Second Affiliated Hospital, Yunnan University of Chinese Medicine, Kunming, China.

Research leader: Taipin Guo

Dear patients,

First of all, thank you for your interest in our clinical study! We would like to invite you to participate in a clinical study on the clinical study of the immediate efficacy of auricular acupuncture combined with active exercise in the treatment of acute lumbar sprains. This study has been approved by the Medical Ethics Committee of the Second Affiliated Hospital of Yunnan University of Chinese Medicine. Before you decide whether or not to participate in this study, please read the following as carefully as possible. It will help you understand the study and why it is being conducted, the procedures and duration of the study, the benefits, risks and discomforts that may be brought to you by participating in the study. If you wish, you can also discuss it with your relatives and friends or ask your doctor to give explanations to help you make your decision.

Study introduction

I. Background and purpose of the study

1. Study background

Acute lumbar sprain is a common clinical condition characterized by persistent intolerable low back pain and limitation of movement, which significantly impacts people's work and life. In

some cases, patients may completely lose their ability to work and perform daily activities. Auricular acupuncture (AA), as a form of traditional acupuncture, offers unique advantages in pain management. Due to its simple treatment modality and rapid onset of action, it is commonly employed in clinical practice for treating acute pain disorders. Previous studies have shown significant pain relief with AA for lumbar pain, but limited efficacy for lumbar mobility disorders. Exercise therapy, commonly utilized in the management of mobility disorders, has shown good efficacy for low back pain when combined with AA, significantly improving pain and function. However, rigorous randomized controlled trials supporting the use of AA combined with exercise therapy for ALS are lacking, necessitating further investigation.

2. Study purposes

The study aims to evaluate the immediate effectiveness of auricular acupuncture combined with active exercise in the treatment of acute lumbar sprains in 10 minutes.

3. Study expected number of participants

This study is expected to include 128 patients with ALS.

II. Who can participate in this study?

(1) Conform to the diagnostic criteria for ALS as outlined in the "Clinical Diagnosis and Treatment Guidelines: Orthopedics" published by the Chinese Medical Association;

(2) Unilateral low back pain, age 18-60 years.

(3) Duration of the disease ≤ 3 days.

(4) Moderate to severe pain, with the visual analogue score (VAS) between 4 and 8.

(5) Signed the informed consent form.

III. Who is not suitable for study?

(1) Combination of lumbar spondylolisthesis, lumbar spine tumor, fracture and other diagnosed definite pathological changes of the lumbar spine.

(2) Low back pain caused by internal medicine diseases.

(3) Coexistence of cardiovascular, hepatic, renal, pulmonary, and hematopoietic systems and

other serious primary diseases.

(4) Severe mental illness or intellectual disability, unable to cooperate with the completion of the questionnaire.

(5) Women in pregnancy or breastfeeding.

(6) Fear of acupuncture, or contraindications to acupuncture such as skin infection at the acupoint site.

(7) Use of other analgesics within the past 6 hours.

IV. What will be done if you participate in the study?

If you meet the inclusion criteria and agree to participate, you will first need to undergo relevant tests to check that you meet all the requirements to participate in the study.

1. Before you are included in the study, you will undergo the following tests to determine if you can participate in the study.

(1) Your medical history, clinical signs and symptoms will be interviewed and recorded; You will also be instructed to complete a Visual Analogue Scale (VAS) score and measure your Range of Motion (ROM) score to determine if you meet our study inclusion criteria.

2. If you meet the inclusion criteria through the above screening, the study will be conducted according to the following steps:

(1) The trial will be divided into 2 groups. At the beginning of the study, your doctor will decide which group you will receive based on the random numbers provided by the computer.

(2) Based on your group allocation, you will undergo a corresponding acupuncture treatment for a duration of 10 minutes. During acupuncture, you will be required to perform moderate low back exercises and cooperate with us in data collection.

3. Other matters requiring your cooperation

In the course of treatment, you need to cooperate with the doctor to complete the relevant scale to fill in, truthfully answer the questions asked by the doctor, cooperate with the doctor, and provide feedback on your condition.

V. Possible benefits of participating in the study

You may benefit from this study, including the possibility of improvement in your condition and

receiving health education about the prevention and treatment of ALS.

VI. Adverse reactions, risks and protective measures for participating in the study

You may have soreness, numbness, heaviness and swelling during the acupuncture process, which are all normal reactions to acupuncture. There may be adverse reactions after needling, but they are rare and mild. You may feel dizzy during needling due to your physical condition or emotional stress, which can be relieved after stopping needling and taking proper rest; bleeding and hematoma may occur after needling, which will disappear after local pressure; however, if infection occurs at the site of needling, your doctor will deal with it promptly.

If you experience any discomfort, new changes in your condition, or any unforeseen circumstances during the study period, whether or not they are related to the acupuncture treatment, you should inform your doctor promptly and he/she will make a judgement and give appropriate medical treatment.

VII. Treatment options available to you other than participating in this study

Your doctor will discuss with you the other treatment options currently available for your condition, including the corresponding risks and benefits. For ALS, there are currently anti-inflammatory and analgesic drugs, mainly non-steroidal anti-inflammatory drugs (NSAIDs), which are effective but have side effects such as gastrointestinal bleeding, gastric ulcers and cerebrovascular accidents.

VIII. The relevant costs

All the costs of this project are supported by the “Liang Fanrong Expert Workstation” of Yunnan Province-Yunnan Provincial Science and Technology Plan Project (202305AF150072), the Youth Special of Yunnan Province Ten-thousand Plan (YNWR-QNBJ-2019-257), and the “Liu Zili Famous Doctor” special talent program of the Yunnan Provincial Xing Dian Talent Support Program (Yunnan Party Talent Office [2022] No. 18). If you participate in this study, you will receive free acupuncture treatment during the study period. This study will only observe the efficacy of the treatment once, and if the subsequent relief is not obvious, you can have 2 free acupuncture treatments. Doctors will make every effort to prevent and treat any harm that may

occur as a result of this study. If an adverse event occurs during the clinical trial, a committee of medical experts will determine whether it is related to the acupuncture treatment or the study process. The sponsor will provide the cost of treatment and financial compensation for any harm related to the trial process in accordance with the provisions of China's Code of Practice for the Quality Management of Pharmaceutical Clinical Trials.

During the treatment period, if you have a combination of other medical conditions, the treatment and examination will not be free of charge.

IX.The confidentiality of clinical data

Your medical records (study charts/CRFs, etc.) will be kept intact at the hospital where you are seen. The investigator, ethics committee and drug regulatory authorities will be given access to your medical records. Any public reporting of the results of this study will not disclose your personal identity. We will make every effort to protect the privacy of your personal medical information to the extent permitted by law.

X. You can voluntarily choose to participate in study and withdraw from the study

Whether or not to participate in the study is entirely up to you. You may decline to participate in the study or withdraw from the study at any time during the study, which will not affect your relationship with the doctor and will not affect your medical or other benefits.

In your best interest, your doctor or researcher may discontinue your participation in this study at any time during your study. If you withdraw from the study for any reason, you may be asked why you are withdrawing.

Your physician will promptly notify you if an important subject-related event or information occurs during the course of the study that may affect your willingness to continue participating in the study.

XI. What should I do now?

Participation in this clinical study is based on a completely voluntary principle and needs to be carried out with your consent and signed informed consent. Whether or not you participate in this clinical study depends entirely on your wishes. You have the right to suspend and withdraw

from this study treatment at any time. Exiting this study will not affect your medical treatment. Your physician may suspend your participation in this study in advance if: Your health condition is not suitable for continued participation, or you may not comply with the study program requirements.

The doctor will promptly notify you or your legal representative if there is medical information that may affect your willingness to continue your study during the study. Before you decide to participate in this study, please ask your life as much as possible until you fully understand this test study.

If you have any questions, suggestions or complaints about this study, please do not hesitate to discuss them with the research team, whose contact details can be found on the signature page. If you feel inconvenienced to communicate with the research team, you can consult or complain to the Medical Ethics Committee of the Second Affiliated Hospital of Yunnan University of Traditional Chinese Medicine. Ethics Committee contact number:15125208547.

Thank you for reading the above material. If you decide to take part in this study, please let your doctor know and he/she will make all the arrangements for you to study.

Informed Consent

Signature Page

1. I have carefully read the contents of the informed consent form, and the researchers have answered my questions.

2. Having fully understood the purpose, methods, possible therapeutic benefits and risks to be encountered and other terms of this clinical study as mentioned in the informed consent form, I voluntarily participate in this study and promise to cooperate fully with the investigators.

3. I understand that I can withdraw from the study at any time and I do not need any reason. The medical services I receive and the legal rights I enjoy are not affected at all.

Finally, I decided to agree to participate in this study and to ensure compliance with my doctor's advice.

Subject Signature: _________________ Date: _________________

Contact Number: _________________

I have explained fully detail to the subjects, including the potential risks.

Doctor/Researcher Signature: _________________ Date: _________________

Contact Number: _________________

知情同意书

告知页

项目名称：耳针结合主动运动治疗急性腰扭伤即时疗效的临床研究

项目来源：云南省科技计划项目-云南省梁繁荣专家工作站（202305AF150072）、云南省万人计划青年拔尖人才项目（YNWR-QNBJ-2019-257）、云南省兴滇英才支持计划"刘自力名医"专项（云党人才办[2022]18号）。

课题研究单位：云南中医药大学针灸推拿康复学院/第二附属医院

项目负责人：郭太品

亲爱的患者：

首先，感谢您对我们这项临床研究的关注！我们将邀请您参加一项"耳针结合主动运动治疗急性腰扭伤即时疗效的临床研究"。本研究已通过云南中医药大学第二附属医院医学伦理委员会审核，同意进行临床研究。在您决定是否参加这项研究之前，请尽可能仔细阅读以下内容。它可以帮助您了解该项研究以及为何要进行这项研究，研究的程序和期限，参加研究后可能给您带来的益处、风险和不适。如果您愿意，您也可以和您的亲属、朋友一起讨论，或者请医生给予解释，帮助您做出决定。

研究介绍

一、研究背景和目的

1. 研究背景

急性腰扭伤是一种常见的临床症状，主要表现为持续性难以忍受的腰部疼痛和活动受限，严重影响人们的工作和生活。在某些情况下，患者可能完全丧失工作和日常活动能力。耳针作为传统针灸的一种，在疼痛治疗方面具有独特的优势。由于其治疗方式简单、起效迅速，临床上常用于治疗急性疼痛疾病。以往的研究表明，耳针疗法对腰部疼痛有明显的缓解作用，但对腰部活动障碍的疗效有限。运动疗法是治疗活动障碍的常用方法，在与耳针联合使用时，对腰痛有良好疗效，可显著改善疼痛和功能。然而，目前还缺乏严格的随机对照试验来支持将耳针与运动疗法相结合治疗急性腰扭伤，因此有必要进行进一步研究。

2. 研究目的

这项研究的目的是评估耳针结合主动运动治疗急性腰扭伤10分钟内的即时疗效。

3. 研究预计纳入参试者例数

本研究预计纳入 128 例急性腰扭伤患者。

二、哪些人能参加这项研究？

1.符合以下条件的人，将会被邀请参加这项研究：

（1）符合中国医学会发布的《临床诊疗指南：骨科分册》中急性腰扭伤的诊断标准；

（2）单侧腰痛，年龄 18-60 岁；

（3）病程≤3 天；

（4）腰部疼痛为中至重度（VAS4-8 分）；

（5）签署知情同意书；

三、哪些人不宜参加本研究

（1）合并腰椎间盘突出症、腰椎肿瘤、骨折及其他经诊断明确的腰椎病理改变者；

（2）内科疾病引起的腰痛者；

（3）合并心血管、肝、肾、肺、造血系统等严重原发性疾病者；

（4）有严重精神疾病或智力障碍，不能配合填写问卷者；

（5）妊娠期或哺乳期妇女；

（6）害怕针灸或有针刺禁忌症者，如穴位部位皮肤感染或破损；

（7）在过去 6 小时内使用过其他镇痛方法者。

四、如果参加研究将要做什么?

1. 在您入选研究前：

医生将询问并记录您的病史、临床症状和体征；医生还将指导您填写腰痛强度视觉模拟量表评分，并测量您的腰部活动范围，以确定您是否符合纳入的标准。

2. 若您通过以上筛查符合纳入标准，将按以下步骤进行研究

（1）试验将分为 2 组。在研究开始时，你的医生将根据电脑提供的随机数决定你将接受哪一组。

（2）根据您的分组信息，您将接受相应的针灸治疗，治疗时间为 10 分钟。在留针期间，您需要进行适度的腰部运动，并配合我们采集数据。

3. 需要您配合的其他事项

在治疗过程中，您需要配合医生完成相关量表填写，如实回答医生提问的问题，配合医

生，对您病情进行反馈。

五、参加研究可能的受益

您将可能从本项研究中受益。此种受益包括您的病情有可能获得改善，以及对您进行预防急性腰扭伤发生的健康教育。

六、参加研究可能的不良反应、风险和不适、不方便

针刺过程中您可能会有酸、麻、重、胀的感觉，这均为针刺的正常反应。针刺后可能存在不良反应，但较少而轻微，针刺时可能因为您的体质问题或情绪紧张出现晕针现象，停止针刺和适当休息后可缓解；针刺后可能出现出血、血肿等现象，经局部按压后可消失；但如果针刺部位出现感染，您的医生会及时处理。

如果在研究期间您出现任何不适，或病情发生新的变化，或任何意外情况，不管是否与针刺治疗有关，均应及时通知您的医生，他/她将对此作出判断并给与适当的医疗处理。

七、除参加本研究外，您可选的其他治疗

您的医生将与您讨论目前针对您的病情可选择的其他治疗方案，包括相应的风险和益处。针对急性腰扭伤的患者，目前可以选择非甾体类抗炎药为主的消炎镇痛药进行治疗，作用效果较好，但是存在胃肠道出血、胃溃疡、脑血管意外等副作用。

八、有关费用

本课题所有费用由云南省科技计划项目-云南省梁繁荣专家工作站（202305AF150072）、云南省万人计划青年拔尖人才项目（YNWR-QNBJ-2019-257）、云南省兴滇英才支持计划“刘自力名医”专项（云党人才办[2022]18 号）项目资助。如您参加本研究，在研究期间，将得到相关免费针刺治疗。本研究仅观察治疗一次的疗效，如后续疗效缓解不明显，可免费做 2 次针刺治疗。医生将尽全力预防和治疗由于本研究可能带来的伤害。如果在临床试验中出现不良事件，医学专家委员会将会鉴定其是否与针刺治疗或研究过程有关。申办者将按照我国《药物临床试验质量管理规范》的规定对与试验过程中相关的损害提供治疗的费用及相应的经济补偿。

在治疗期间，如果您同时合并其他疾病所需的治疗和检查，不在免费的范围之内。

九、个人信息是保密的吗？

您的医疗记录（研究病历/CRF 等）将完整地保存在您所就诊的医院。研究者、伦理委员会和药品监督管理部门将被允许查阅您的医疗记录。任何有关本项研究结果的公开报告将不会披露您的个人身份。我们将在法律允许的范围内，尽一切努力保护您个人医疗资料的隐私。

十、可以自愿选择参加研究和中途退出研究

是否参加研究完全取决于您的意愿。您可以拒绝参加此项研究，或在研究过程中的任何时间退出本研究，这都不会影响您和医生间的关系，都不会影响您的医疗待遇与权益，或有其他方面利益的损失。

出于对您的最大利益考虑，医生或研究者可能会在研究过程中随时中止您继续参加本项研究。如果您因为任何原因从研究中退出，您可能被询问有关您接受针灸或药物治疗的情况。

研究过程中如果发生与受试者相关的重要事件或信息，可能会影响您继续参加研究的意愿时，您的医生将及时通知您。

十一、怎样获得更多的信息?

参加本项临床研究，本着完全自愿的原则，需要在您同意并签署知情同意书的前提下进行。是否参加本项临床研究，完全取决于您本人的意愿，您有权在任何时候选择中止和退出本项研究性治疗，退出本研究并不会影响您的医疗待遇。

您的医师可以在下列情况下提前中止您继续参加本项研究：您的健康状况不适合继续参加，或者您不能遵守研究方案的要求。

如在研究过程中出现可能影响您继续参加研究意愿的医学信息，医生将及时通知您或者您的法定代表。在您做出参加本研究的决定前，请尽可能向您的医生询问有关问题，直至您对本项试验研究完全理解。

如您对这项研究存在任何疑问、建议或投诉，请及时与研究团队讨论，联系方式见签字页。如您感觉不便与研究团队沟通，可向云南中医药大学第二附属医院医学伦理委员会进行咨询或投诉。伦理委员会联系电话:13888244951。

感谢您阅读以上材料。如果您决定参加本项研究，请告诉您的医生， 他/她会为您安排切有关研究的事务。

知情同意书

签字页

1. 我已经仔细阅读了知情同意书告知页的内容，研究者已解答了我提出的疑问。

2. 我在充分理解了知情同意书提及的本项临床研究的目的、方法、可能获得的治疗利益和可能遇到的风险以及其他条款后，自愿参加此项研究，并承诺与研究者充分合作。

3. 我明白我可在任何时候退出研究，并且不需要任何理由，我得到的医疗服务和享有的法律权利不受任何影响。

最后，我决定同意参加本项研究，并保证遵从医嘱。

受试者签名:________________　　　　日期：_______年_______月_______

联系电话:___________________

我确认已向患者解释了本研究的详细情况，包括其权力及可能的受益和风险。

医生/研究者签名:____________　　　　日期：_______年_______月________

联系电话:___________________
